# Factors Associated with Functional Limitation in Stair Climbing in Female Japanese Patients with Knee Osteoarthritis

**DOI:** 10.2188/jea.16.21

**Published:** 2005-12-20

**Authors:** Kyoko Kondo, Takashi Tanaka, Yoshio Hirota, Hideya Kawamura, Hiromasa Miura, Yoichi Sugioka, Hajime Inoue, Masahiro Kurosaka, Terumi Yamashita, Kumiko Shirata, Yukihide Iwamoto

**Affiliations:** 1 Department of Public Health, Osaka City University Faculty of Medicine.; 2 Department of Orthopaedic Surgery, Kyushu University Faculty of Medicine.; 3 Department of Orthopaedic Surgery, Okayama University Faculty of Medicine.; 4 Department of Orthopaedic Surgery, Kobe University Faculty of Medicine.; 5 College of Nursing, Osaka City University.

**Keywords:** Osteoarthritis, functional limitation, stair climbing, Body Weight, Parity

## Abstract

**BACKGROUND:**

Osteoarthritis (OA) of the knee is a common form of arthritis, and affects quality of life. We investigated factors associated with functional limitation in stair climbing among female Japanese patients with knee OA. As weight is a known risk factor for knee OA, we focused on body weight at 40 years of age, and examined the association with present weight, past weight, and weight change.

**METHODS:**

Subjects were 360 Japanese women aged 40-92 years who were newly diagnosed with knee OA at 3 university hospitals over a 1-year period. Factors associated with the severity of functional limitation in stair climbing were assessed by calculating odds ratios (OR) using the proportional odds model in logistic regression.

**RESULTS:**

Weight at diagnosis showed a positive association with severe functional limitation in stair climbing; however, a negative association was observed for weight change since age 40. Further analysis indicated that the association with weight at age 40 (highest vs. lowest quartile, OR=2.84, 95% confidence interval: 1.03-7.83, trend p=0.071) is stronger than weight at diagnosis. Other significant characteristics were age (70+ vs. 40-59 years, OR=7.37), previous knee pain and/or swelling 12 years or more before diagnosis (OR=2.67), and physical work (OR=1.94). In addition, higher parity was found to be a negatively associated factor (for tripara or more, OR=0.41).

**CONCLUSIONS:**

This study identified factors, such as heavy weight at age 40 and physical labor, which are potentially useful for preventing severe functional limitation for female knee OA patients. In addition, higher parity was associated with milder stair climbing limitation.

Arthritis is a major chronic disease in the United States, with the total number of self-reported arthritis cases expected to increase from 43 million in 1997 to 60 million in 2020.^1^^,^^2^ Osteoarthritis (OA) of the knee is a common form of arthritis in the elderly. One recent study in the United States reported the age- and sex-standardized incidence rate for knee OA as 240/100,000 person-years, with the incidence of knee OA increasing with age and with a high incidence occurring in women after 50 years of age.^3^ One study in Japan estimated the annual incidence of knee OA at approximately 900,000, with 87% of the patients older than 50 years.^4^ As incidence of knee OA is expected to rise as the proportion of elderly population continues to increase, and knee OA has a substantial impact on activities of daily living, several epidemiologic studies have investigated risk factors for knee OA, finding a consistent association between the incidence or progression of knee OA and age,^5^^,^^6^ obesity,^5^^-^^12^ weight change,^13^^,^^14^ history of knee injury,^5^^,^^10^^,^^12^^,^^15^ occupational physical demands,^7^^,^^12^ occupational exposure,^15^ physical activity,^14^^,^^16^ and regular sports activities.^10^^,^^15^ However, reported relationships with nutritional factors and other systemic factors, such as serum C-reactive levels, diabetes, and hypertension, remain controversial.^17^ Furthermore, few OA studies have been conducted in Japan for knee^4^^,^^6^^,^^12^ or other forms of OA.^18^^,^^19^

In 1999, a study of the prevalence of disabilities and associated health conditions among adults in the United States revealed that 50% of adults aged 65+ years had a disability, and a high percentage of adults aged 65+ years reported difficulty with the following specific functional activities: climbing a flight of stairs (30.4%) and walking three city blocks (30.5%). Of the main health conditions associated with these disabilities, arthritis and rheumatism were the most common (17.5%).^20^ The Framingham Study demonstrated that knee OA did not affect all types of functional activity equally in the elderly.^21^ Another research has reported that knee OA is associated with limitation in stair climbing after adjustments for age, sex, and comorbidity; furthermore, the proportion of limitation in stair climbing associated with knee OA is substantially greater than the proportion attributable to heart disease and other medical conditions.^22^ As stair climbing reflects muscle strength (knee torques), we analyzed functional limitation in stair climbing as an outcome and examined its association with anthropometric measurements and patient characteristics. In previous research, dependence on human assistance has been used as a criterion of functional limitation on walking up and down stairs.^21^^,^^22^ Therefore, we established a “Help on stairs” criterion modified from the Assessment Criteria of Treatment for knee OA of the Japanese Orthopaedic Association.^23^ Although weight is a known risk factor for knee OA, examining the association using only present weight will not allow us to refute the hypothesis that prior knee OA results in marked weight gain due to reduced mobility. Therefore, we focused on body weight at age 40, because of the higher prevalence of knee OA after age 40, and examined whether heavy weight precedes knee OA or vice versa.

## METHODS

### Study Subjects

Subjects included all patients newly diagnosed with knee OA at 3 Japanese hospitals (Okayama University Hospital, Kobe University Hospital, and Kyushu University Hospital) between October 1, 1991 and September 30, 1992. Diagnoses were made according to the clinical and radiographic definition of the American College of Rheumatology,^24^ the criteria of which includes knee pain, osteophytes, and 1 of the following: 50+ years of age, morning stiffness <30 minutes duration, or crepitus on active motion of the knee.

A total of 608 identified patients were asked to complete a self-administered questionnaire at the time of diagnosis and return it by mail to each university hospital. The questionnaire was designed to elicit information regarding pain, functional limitation, and associated factors. The response rate was 82% (497/608; 116 male, 367 female, 14 sex not indicated). After excluding females younger than 40 years of age (7 patients), male patients (116 patients), and responses that did not indicate sex (14 patients), we analyzed the remaining 360 female cases.

### Data Collection

To assess the functional limitation in stair climbing associated with knee OA, subjects responded to a question modified from the Assessment Criteria of Treatment for Knee OA of the Japanese Orthopaedic Association:^23^ “Do you need help on stairs?”, which referred to the need for aids (such as a handrail) or assistance while climbing and/or descending stairs (responses: always necessary, sometimes necessary, unnecessary).

Data collected about factors associated with functional limitation in stair climbing were as follows: date of birth, height and weight at diagnosis (obtained from medical record), weight at age 40, previous knee pain and/or swelling with no relation to the present symptoms (yes, no, age at manifestation if yes), history of knee injury requiring medical treatment for 2+ weeks (yes, no), history of injury to lower extremities (except knee) requiring medical treatment for 2+ weeks (yes, no), history of other conditions requiring medical treatment for 3+ months (yes, no, details if yes), good posture while sitting (sitting on the floor Japanese style, with knees bent and toes directly beneath the body) (no, sometimes, often), smoking (never, past, current), alcohol consumption (never, past, current), sports participation such as club activities in school (yes, no, duration if yes), occupation of greatest duration (open-ended question), education (junior high school, high school, junior college or higher), and parity (age at first delivery and the number of deliveries if parous).

### Statistical Analysis

We analyzed the responses to “Do you need help on stairs?” (always necessary, sometimes necessary, unnecessary) as an outcome index.

For explanatory variables, occupations were categorized according to the classifications of the Japan National Census and divided into two groups: physical workers were defined as “agricultural/fishery workers”, “mining workers”, and “factory/construction workers or laborers”; all other occupations were defined as nonphysical workers. Previous knee pain and/or swelling with no relation to the present symptoms was categorized into three categories (none; recent: within past 12 years; past: 12+ years before) for comparison in accordance with the median of 12 years if yes. Continuous variables were categorized for comparison in accordance with the approximate quartile except for age (40-59/ 60-69/ 70+) and weight change (decreased: <0/ almost no change: 0-5/ increased: 6+ kg) because almost one third of subjects were distributed in their sixties for age and in the decreased weight category.

To express the associations between severe functional limitation in stair climbing and anthropometric and other characteristics, odds ratios (ORs) and their 95% confidence intervals (CIs) were computed using the proportional odds model^25^^-^^27^ in logistic regression. We calculated the p-values for the score to test for the proportional odds assumption in order to confirm that use of the proportional odds models would be appropriate for the models. The test for trend was performed by including in the model explanatory variables that were coded by ordinal numbers with increasing exposure categories. Student’s t-test, Wilcoxon rank sum test, Kruskal-Wallis test, Chi-square test, and Spearman rank correlation coefficients were also used where appropriate.

To construct the multivariate models, the explanatory variables were selected as follows: (1) calculating the crude ORs in univariate analysis and the age-adjusted ORs in multivariate analysis, and (2) conducting multivariate analysis involving the variables which showed statistically significant crude or age-adjusted ORs, as well as important pathophysiological variables whether statistically significant or insignificant.

All analyses were performed with Statistical Analysis System^®^ Version 9.1 (SAS Institute, Inc., Cary, NC, USA).

## RESULTS

Table 1 shows selected subject characteristics. Among all the subjects at diagnosis, 86.7% indicated that they always or sometimes needed help on stairs.

**Table 1.** Selected subject characteristics.

Characteristics*		No. of subjects	Mean or Percent
Mean age (year)	(n=367)		65.1
Mean height at diagnosis (cm)	(n=358)		151.5
Mean weight at diagnosis (kg)	(n=361)		56.2
Mean weight at age 40 (kg)	(n=343)		53.6
Mean weight gain (kg)	(n=342)		2.5

Previous knee pain and/or swelling	(n=345)		
none		145	
past (mean years: diagnosis- previous) (years)		200	15.1

History of medical treatment (3+ months)	(n=345)	214	62.0%

Smoking	(n=352)		
never		295	83.8%
past		28	8.0%
current		29	8.2%

Habitual alcohol consumption	(n=329)		
never		273	83.0%
past		14	4.3%
current		42	12.8%

Education	(n=367)		
junior high school		98	26.7%
high school		185	50.4%
junior college or higher		84	22.9%

Mean number of deliveries (times)	(n=361)		2.5

Help on stairs	(n=353)		
always necessary		157	44.5%
sometimes necessary		149	42.2%
unnecessary		47	13.3%

* : Variables are expressed as percent, unless otherwise specified.

We included 10 explanatory variables in the multivariate models (Table 2): age at diagnosis, height and weight at diagnosis, weight change since age 40 or weight at age 40, previous knee pain and/or swelling with no relation to the present symptoms, history of medical treatment, smoking, sports participation in school, occupation, and parity. Age at first delivery showed statistically significant OR in univariate analysis, but parity was adopted for the explanatory variable to avoid the elimination of nulliparous women from the model.

**Table 2.** Association between selected characteristics and functional limitation in stair climbing of newly-diagnosed knee osteoarthritis patients.

Characteristics	Help on stairs			Univariate		Multivariate model 1*^‡^		Multivariate model 2^†‡^	

	unnecessary	sometimes necessary	always necessary						
			
	n (%)	n (%)	n (%)	OR (95% CI)	p value	OR (95% CI)	p value	OR (95% CI)	p value
Age (year)
40-59	25 (54)	50 (34)	27 (18)	1		1		1	
60-69	14 (30)	67 (46)	60 (39)	2.29 (1.40-3.74)	0.001	1.59 (0.84-3.00)	0.152	1.75 (0.94-3.29)	0.080
70+	7 (15)	29 (20)	67 (44)	5.34 (3.06-9.34)	0.000	6.81 (3.14-14.8)	0.000	7.37 (3.39-16.0)	0.000
				(Trend: p= 0.000)		(Trend: p= 0.000)		(Trend: p= 0.000)	

Weight at diagnosis (kg)
-50	13 (29)	43 (30)	34 (23)	1		1		1	
51-55	12 (27)	40 (28)	33 (22)	1.04 (0.59-1.82)	0.894	1.28 (0.62-2.67)	0.504	0.82 (0.38-1.76)	0.604
56-60	9 (20)	30 (21)	40 (26)	1.59 (0.89-2.83)	0.115	1.44 (0.67-3.11)	0.348	0.80 (0.35-1.82)	0.594
61+	11 (24)	32 (22)	44 (29)	1.55 (0.88-2.72)	0.126	2.58 (1.14-5.84)	0.023	1.12 (0.45-2.80)	0.805
				(Trend: p= 0.058)		(Trend: p= 0.026)		(Trend: p= 0.840)	

Weight change since age 40 (kg)
<0	10 (23)	42 (30)	53 (37)	1		1			
0-5	23 (53)	52 (37)	49 (34)	0.59 (0.36-0.96)	0.036	0.44 (0.23-0.83)	0.012	-	
6+	10 (23)	47 (33)	43 (30)	0.78 (0.46-1.32)	0.355	0.46 (0.22-0.93)	0.031	-	
				(Trend: p= 0.353)		(Trend: p= 0.028)			

Weight at age 40 (kg)
-47	13 (30)	32 (23)	28 (19)	1				1	
48-52	10 (23)	43 (31)	41 (28)	1.37 (0.77-2.44)	0.292	-		2.05 (0.94-4.43)	0.070
53-59	10 (23)	34 (24)	32 (22)	1.24 (0.68-2.27)	0.489	-		2.07 (0.87-4.93)	0.101
60+	10 (23)	32 (23)	45 (31)	1.75 (0.96-3.16)	0.066	-		2.84 (1.03-7.83)	0.044
				(Trend: p= 0.103)				(Trend: p= 0.071)	

Height (cm)
-148	10 (22)	33 (23)	50 (33)	1		1		1	
149-151	7 (16)	37 (26)	29 (19)	0.66 (0.36-1.18)	0.158	0.78 (0.37-1.65)	0.518	0.72 (0.34-1.53)	0.398
152-155	15 (33)	42 (29)	34 (23)	0.53 (0.30-0.92)	0.024	1.03 (0.49-2.17)	0.939	0.98 (0.46-2.10)	0.959
156+	13 (29)	31 (22)	38 (25)	0.71 (0.40-1.25)	0.234	1.39 (0.64-3.04)	0.411	1.26 (0.56-2.80)	0.579
				(Trend: p= 0.143)		(Trend: p= 0.394)		(Trend: p= 0.583)	

Previous knee pain and/or swelling
none	31 (69)	53 (38)	54 (37)	1		1		1	
recent (within past 12 years)	7 (16)	54 (39)	36 (25)	1.31 (0.80-2.14)	0.282	1.44 (0.81-2.57)	0.219	1.41 (0.79-2.53)	0.245
past（12+ years before)	7 (16)	31 (22)	56 (38)	2.68 (1.60-4.50)	0.000	2.83 (1.45-5.55)	0.002	2.67 (1.37-5.19)	0.004
				(Trend: p= 0.000)		(Trend: p= 0.003)		(Trend: p= 0.004)	

History of medical treatment (3+ months)
no	21 (54)	55 (40)	49 (33)	1		1		1	
yes	18 (46)	84 (60)	99 (67)	1.62 (1.06-2.48)	0.027	1.21 (0.71-2.06)	0.484	1.27 (0.75-2.17)	0.374

Smoking
never	41 (91)	121 (86)	119 (80)	1		1		1	
past	3 (7)	7 (5)	17 (11)	2.17 (0.98-4.80)	0.056	1.22 (0.45-3.34)	0.699	1.21 (0.45-3.28)	0.705
current	1 (2)	12 (9)	13 (9)	1.55 (0.71-3.37)	0.270	1.45 (0.51-4.12)	0.485	1.77 (0.61-5.08)	0.292
				(Trend: p= 0.093)		(Trend: p= 0.444)		(Trend: p= 0.279)	

Sports participation in school
no	26 (62)	88 (66)	104 (73)	1		1		1	
yes	16 (38)	46 (34)	38 (27)	0.68 (0.44-1.07)	0.095	0.64 (0.35-1.15)	0.132	0.68 (0.38-1.22)	0.198

Occupation
non-physical worker	32 (73)	113 (81)	96 (68)	1		1		1	
physical worker	12 (27)	27 (19)	46 (32)	1.56 (0.97-2.52)	0.066	1.95 (1.03-3.67)	0.040	1.94 (1.03-3.69)	0.042

Parity (times)
0, 1	4 (9)	30 (21)	27 (18)	1		1		1	
2	22 (49)	44 (31)	45 (31)	0.68 (0.38-1.23)	0.204	0.53 (0.24-1.15)	0.108	0.51 (0.23-1.11)	0.089
3+	19 (42)	69 (48)	75 (51)	0.96 (0.55-1.68)	0.886	0.42 (0.20-0.88)	0.021	0.41 (0.19-0.86)	0.019
				(Trend: p= 0.784)		(Trend: p= 0.026)		(Trend: p= 0.025)	

OR: odds ratio, CI: confidence interval

* : Model 1 comprises age, height and weight at diagnosis, weight change since age 40, previous knee pain and/or swelling, history of medical treatment, smoking, sports participation in school, occupation, and parity as explanatory variables.

† : Model 2 comprises age, height and weight at diagnosis, weight at age 40 years, previous knee pain and/or swelling, history of medical treatment, smoking, sports participation in school, occupation, and parity as explanatory variables.

‡ : Analysis based on a sample size of 258.

Of the 360 subjects, 258 completed the questionnaire for the multivariate analysis (perfect responders), but 102 were missing some data (imperfect responders) (Table 3). Missing data was relatively frequent for previous knee pain and/or swelling, history of medical treatment, sports participation in school, and occupation, although it was seen in less than 5% of the subjects for other variables. Because potential bias due to imperfect responders should be considered, we compared selected characteristics between the perfect and imperfect responders. The imperfect responders, as compared to perfect responders, were older, weighed less at diagnosis, were shorter, experienced more medical treatment, and had a higher parity. Second, we examined correlations among these five variables. The correlation coefficients with age were: -0.300 (p=0.000) for height, -0.160 (p=0.003) for weight at diagnosis, 0.172 (p=0.002) for history of medical treatment, and 0.327 (p=0.000) for the number of deliveries.

**Table 3.** Comparison of selected characteristics between perfect and imperfect responders.

Characteristics*	No. missing data	Mean or Percent		p value

		perfect responders^†^ (n=258)	imperfect responders (n=102)	
Mean age (year)	0	64.6	68.7	0.000^‡^
Mean weight at diagnosis (kg)	6	56.4	55.2	0.047^§^
Mean weight gain (kg)	18	2.8	1.4	0.225^§^
Mean weight at age 40 (kg)	17	53.6	53.3	0.824^§^
Mean height at diagnosis (cm)	9	151.8	150.5	0.053^§^
Previous knee pain and/or swelling	22	57.4	61.3	0.189^∥^
History of medical treatment (3+ months)	22	60.1	71.3	0.071^∥^
Current smoking	15	7.8	10.3	0.744^∥^
Sports participation in school	32	32.6	27.1	0.387^∥^
Physical worker	23	24.4	29.1	0.402^∥^
Mean number of deliveries (times)	13	2.5	3.0	0.005^§^
Always needs aid or assistance while climbing and/or descending stairs	14	42.3	51.1	0.282^∥^

* : Variables are expressed as percent, unless otherwise specified.

† : Outcome is the same as that of Table 2.

‡ : Student’s t-test, §: Wilcoxon rank sum test, ∥: Chi-square test

Table 2 shows the association between the selected characteristics and functional limitation in stair climbing of newly diagnosed knee OA patients. In Model 1, weight at diagnosis and weight change since age 40 were included as weight variables. Before the proportional odds model was adapted to Model 1, we calculated the p-values for the score to test for the proportional odds assumption (p=0.245). Thus we interpreted that use of the proportional odds models would be appropriate. Older age was associated with greater OR with a clear dose-response relation, and OR of those aged 70 or older reached a statistically significant level. For body weight at diagnosis, elevated ORs were observed at the fourth quartile showing a significant dose-response. For the effect of weight change since age 40, ORs for weight gain decreased. This negative association was also statistically significant. Decrease in crude ORs for the upper categories of height seen in the univariate analysis may be due to the correlation of younger age and greater height. Such a finding for height was no longer evident in multivariate analysis. Previous knee pain and/or swelling showed significant increase in OR if it had occurred 12 years or more before diagnosis of knee OA, but there was no significant increase for occurrence within the past 12 years. History of medical treatment was correlated with functional limitation in stair climbing in the univariate analysis, but no statistically significant OR was obtained when considered with other factors. Physical workers showed two-fold greater OR as compared to non-physical workers. Multipara was associated with decreasing ORs with significant results in the test for trend, showing a significant decrease in tripara or more.

Severity of stair climbing limitation was associated positively with body weight at diagnosis, but negatively with weight gain since age 40. These apparent contradictory findings suggest that subjects with a lower weight at age 40 were more likely to be classified as low weight at diagnosis regardless of weight gain, and conversely, overweight subjects at age 40 might still be classified at a heavy weight at diagnosis in spite of weight loss. We then performed an additional calculation in the slightly modified Model 1 in which weight change was replaced by weight at age 40 (Model 2). Because we calculated a p-value of 0.249 when testing for the proportional odds assumption, we determined that use of the proportional odds model in Model 2 was appropriate. Elevated ORs for weight at age 40 were obtained, but ORs for weight at diagnosis were no longer significant. The other significant variables, such as age, previous knee pain with no relation to the present symptoms, occupation, and parity, had ORs with the same direction and similar pattern as in Model 1. In Model 2, when weight and height at diagnosis and weight at age 40 were replaced by body mass index (BMI) (kg/m^2^) at diagnosis and BMI at age 40, ORs calculated for both BMIs became ambiguous. Subsequently, we adopted Model 2 as the final model.

Next, we examined the mutual effects of weight at age 40 and at diagnosis. For this purpose, weight change patterns were categorized into the following four patterns with the cut-off values of median weight at age 40 (52 kg) and at diagnosis (55 kg): subjects with low weight at age 40 and at diagnosis (L-L group), low weight at age 40 but heavy weight at diagnosis (L-H group), heavy weight at age 40 but low weight at diagnosis (H-L group), and heavy weight at age 40 and at diagnosis (H-H group). In the final model, weight at age 40 and at diagnosis were replaced by these weight change patterns (Table 4, Model 3). Use of the proportional odds model in Model 3 was appropriate because a p-value of 0.173 was observed when testing for the proportional odds assumption. Subjects in the L-H group showed a somewhat increased but statistically insignificant OR, as compared to the reference category (L-L group). For subjects with heavy weight at age 40 (H-L group, H-H group), the obtained elevated ORs were marginally significant irrespective of weight at diagnosis when compared to the reference category. It is noteworthy that in the H-L group (n=29), marginally significant increased OR was detected, even though fewer subjects were included in the distribution than the L-H group (n=41).

**Table 4.** Weight change patterns and functional limitation in stair climbing of newly-diagnosed knee OA patients

Weight at age 40 (kg)	Weight at diagnosis (kg)	unnecessary*	sometimes necessary	always necessary	Model 3^†^	
	
		n (%)	n (%)	n (%)	OR (95% CI)	p value
-51.9	-54.9	15 (44)	37 (32)	27 (25)	1	
-51.9	55.0+	4 (12)	19 (17)	18 (17)	1.29 (0.60-2.79)	0.514

52.0+	-54.9	2 (6)	13 (11)	14 (13)	2.18 (0.88-5.37)	0.092
52.0+	55.0+	13 (38)	46 (40)	50 (46)	1.86 (0.98-3.53)	0.057

* : Outcome is the same as that of Table 2.

† : Model included same variables as mentioned in the footnote of model 2 in Table 2 except weight at diagnosis and weight at age 40 years, which were replaced by weight change patterns. Weight at age 40 and at diagnosis were treated as dichotomous variables with a median cut-off value.

## DISCUSSION

Many previous studies have reported a strong association between weight and knee or hip OA.^4^^-^^6^^,^^9^^-^^11^^,^^15^^,^^28^^-^^33^ Some studies based on the Framingham Study have identified certain relationships between weight and OA:^8^^,^^13^^,^^14^ obese persons at baseline more frequently developed knee OA approximately 36 years later, with a two-fold relative risk in the heaviest quintile; weight gain or loss directly correlated with increase or decrease in the risk of developing radiographic knee OA among elderly persons; and weight loss reduced the risk for symptomatic knee OA while weight gain slightly increased the risk with no statistical significance among women. In our analysis, each of three weight-related indices, weight at age 40, weight change since age 40, and weight at diagnosis, were associated with the severity of functional limitation in stair climbing for knee OA patients, but weight at age 40 was the dominant associated factor as shown in Table 2. Further analysis in Table 4 indicated that heavy weight at age 40 is the dominant associated factor irrespective of weight at diagnosis. Because our subjects were newly diagnosed, knee OA should not have occurred for about 25 years after age 40. These findings also may support the notion that heavy weight is a predictor for stair climbing limitation of knee OA patients, and not an outcome of knee OA. Thus it would be reasonable to consider that weight control in early middle age is potentially useful for preventing severe functional limitation in stair climbing for subsequent knee OA. When weight and height were replaced by BMI in the final model, ORs for BMI became obscure. This seems reasonable because BMI is an obesity index that factors in height, which was an insignificant variable in our analysis.

In the present study, previous knee pain and/or swelling 12 years or more before diagnosis was related to stair climbing limitation. The mean period of time from previous knee pain and/or swelling through diagnosis of knee OA was 15.1 years (Table 1). Thus, it is reasonable to consider that this variable is a predictor of stair climbing limitation rather than the prodrome of the present symptoms. However, this variable can be a proxy for other risk factors. For example, joint injury is a well-known risk factor for knee OA,^5^^,^^10^^,^^12^^,^^15^ although we did not observe a relationship between history of knee injury requiring medical treatment for 2+ weeks and stair climbing limitation. Those with moderate condition which was not so severe as they report it to be the history of knee injury might have answered “yes” to the question on the previous knee pain and/or swelling.

The present study showed 7-fold ORs in stair climbing limitation for older subjects (70+ vs. 40-59 years of age) after adjustment for other factors. A previous cross-sectional study reported that age was significantly associated with knee OA in elderly persons.^5^ The increasing incidence and prevalence of knee OA with age may be a consequence of several biologic changes that occur with aging.^17^ Women have a high incidence of knee OA after age 50 and this is the approximate age of menopause. However, we did not have data for menopausal status available to us in the present study. Therefore, we performed an additional analysis for those subjects 55+ years in the final model and found that the same factors had similar results. Hormone replacement therapy is known to be associated with a reduction in the risk of knee OA but the mechanism of the negative relation is unclear.^34^^,^^35^ We did not obtain information on hormone replacement therapy because only 1.2% of women aged 45-64 years were receiving the hormone replacement therapy in Japan.^36^ Thus far, few studies have reported on the relationship between parity and risk of knee OA. We found a clear negative association between higher parity and functional limitation in stair climbing of knee OA patients. The smaller decrease in crude ORs for multipara is attributable to the offsetting at older ages. This negative relationship can be explained by the calcium loss involved in pregnancy, since increased bone mineral density is recognized to be a risk factor for knee OA.^6^^,^^32^ On the other hand, nullipara may be associated with a disturbance in hormonal balance, because nullipara may be infertile, and female infertility is associated with hormonal balance.^37^ Therefore, a negative association between multipara and stair climbing limitation of knee OA patients might be indicative of a trend due to the positive association between nullipara and severe symptoms.

The association of OA with manual occupations has been reported, with regards to intensity, frequency, and duration.^15^^,^^38^ A case-control study in Japan reported that sedentary work during initial employment (OR=0.35, 95% CI=0.15-0.84), and total working years (1.05, 1.01-1.08) were factors associated with knee OA, after controlling for other potential risk factors.^12^ In the present study, subjects who were classified as physical workers tended to show severe limitation in stair climbing. This finding is consistent with a previous cross-sectional study, which showed a positive association between heavy physical demands and knee OA.^7^ Long-term heavy physical occupations or mechanical stress may therefore play a role in the functional limitation in stair climbing of knee OA patients.

There are several limitations in our study. First, with regard to the validity of diagnosis, we did not assess inter-observer and inter-institution variation. However, the diagnostic criteria based on clinical and radiographic findings has 91% sensitivity and 86% specificity, where specificity is higher than criteria based on clinical and laboratory findings (75%) or on clinical findings alone (69%), although sensitivity is similar to the former (92%) and the latter (95%).^24^ Thus, the dilution of subjects with non-OA patients would have been minimal.

As to a second limitation, there is a possibility that responses to “Do you need help on stairs?” reflect aging and other diseases aside from knee OA caused by aging. A previous study of the elderly reported the proportion of dependence in stair climbing as an adjusted attributable fraction for each condition as follows: knee OA (16.7%), depressive symptomatology (15.4%), stroke (12.8%), diabetes (9.8%), hip fracture (9.1%), congestive heart failure (7.6%), and chronic obstructive pulmonary disease (7.4%).^22^ In the present study, details of medical history were as follows: hypertension 77, gastrointestinal disease 47, heart disease 41, tuberculosis 25, diabetes 23, gallstone or gallbladder disease 23, liver disease 19, cerebral apoplexy 2, and other diseases 69. When ORs were calculated in the final model for subjects without each disease, all the significant variables showed similar ORs in terms of direction and pattern. However, the effect of other diseases aside from knee OA cannot be ignored because we did not obtain data regarding depressive symptomatology, hip fracture, congestive heart failure, or obstructive pulmonary disease, which may have affected responses to “Do you need help on stairs?”.

Knee OA was associated with dependence in four tasks (stair climbing, walking one mile, house keeping, and carrying a bundle).^22^ Thus, these three additional tasks must be examined in order to interpret overall functional limitation in knee OA.

When we considered potential bias due to imperfect responders in the multivariate model (Model 1, 2), it can be interpreted that imperfect responders included older subjects, and therefore they were shorter, weighed less, experienced more medical treatment, and had a higher parity. All these variables were simultaneously considered in the final multivariate model.

Finally, recall bias related to self-reported body weight at age 40 may be another potential limitation because we were unable to assess the reliability of recalled body weight at age 40. A longitudinal study confirmed that correlations between recalled weights at ages 18 and 40 when participants were 50 years old and measured weights at ages 18 and 40 were 0.87 and 0.95 respectively.^39^ Therefore, weights at age 40 in our study are assumed to be reliable, although not exact. Furthermore, our study is meaningful, because several studies have reported the association between past weight and stair climbing limitation in knee OA patients.

In summary, we found several factors associated with functional limitation in stair climbing in newly diagnosed knee OA patients. Excessive weight at age 40 was more strongly associated with stair climbing limitation than weight at diagnosis or weight change after age 40. Previous knee pain and/or swelling 12 years or more before diagnosis or the proxy variable for unknown factors, and physical work were also correlated with this limitation, whereas multipara was a factor for milder limitation associated with knee OA.
